# There’s Danger in the Drops: Systemic Effects of Ophthalmic Drops Used to Treat Glaucoma

**DOI:** 10.7759/cureus.20945

**Published:** 2022-01-04

**Authors:** Claire Haga, Thomas Waller, Abhimanyu S Ahuja, Bryan Farford, Nancy Dawson, Mingyuan Yin

**Affiliations:** 1 Family Medicine, Mayo Clinic, Jacksonville, USA; 2 Medicine, Florida Atlantic University Charles E. Schmidt College of Medicine, Boca Raton, USA; 3 Internal Medicine, Mayo Clinic, Jacksonville, USA; 4 Research Administration, Mayo Clinic, Jacksonville, USA

**Keywords:** beta-blockers, internal medicine, family medicine, ophthalmology, side effects, ophthalmologic drops, glaucoma

## Abstract

Glaucoma is a common eye disorder and an irreversible cause of blindness worldwide. There are several treatment options for this condition, with the traditional first-line treatment being ophthalmologic drops. Although administered topically, it is associated with inadvertent systemic absorption leading to a potential for both local and systemic side effects. We discuss the case of a 71-year-old male who presented with a complaint of recurring episodes of distressing sensations including lightheadedness, dyspnea, chest pressure, and faintness. His past medical history included congestive heart failure, hypertension, hyperlipidemia, Barrett’s esophagus, and glaucoma. Upon a thorough review of the patient’s medications, it was discovered that he had recently been started on timolol ophthalmic drops. The patient then noted that his symptoms had begun after he started using the eye drops. After we recommended that the patient hold the use of the eye drops, these episodes stopped. When prescribing topical ophthalmologic drops, providers must educate patients on common systemic side effects of such drugs.

## Introduction

Glaucoma is the second most prevalent cause of permanent blindness in the United States (US), and it is projected that 111.8 million people worldwide will have glaucoma by the year 2040 [1,2]. It is also a leading cause of blindness among African Americans and Hispanics [3,4]. The progressive vision loss associated with glaucoma is due to optic nerve damage [4]. The resulting visual field defects correspond to the location of the optic nerve damage. Mild damage may be asymptomatic, but as the damage worsens, symptoms begin to develop, including patchy loss of peripheral vision. If the elevated intraocular pressure remains untreated, it will often lead to irreversible blindness. Even among patients who undergo treatment, nearly 15% will develop blindness in at least one eye within 20 years [4,5]. As vision loss cannot be reversed, it is imperative to detect and initiate prompt and early treatment. The aim of treatment is to reduce intraocular pressure, either by decreasing the production of aqueous humor or improving outflow through various pathways, thereby protecting the optic nerve and decreasing the progression of vision loss [6]. There are several treatment options, including ophthalmic drops, laser procedures, and surgery. In the US, eye drops are the most frequently employed first-line therapy for the treatment of glaucoma. Laser treatment is similar in efficacy to topical agents. It can be used as an initial or adjunct therapy and is often used in patients with poor medication tolerance or compliance [3,4,6]. Surgical modalities are typically reserved for individuals who demonstrate progressive visual loss despite medical or laser therapy [3,4].

## Case presentation

We report the case of a male patient who presented to an outpatient primary care clinic for symptoms occurring after starting timolol eye drops. A 71-year-old male presented to a Family Medicine clinic with two weeks of recurrent, distressing sensations that he described as abrupt feelings of lightheadedness spreading from the top of his head, down his face, and into his chest, with each episode lasting about 15 seconds. During these episodes, he experienced shortness of breath, chest pressure, and faintness. His wife added that during the episodes he appeared pale. His past medical history included congestive heart failure, hypertension, hyperlipidemia, Barrett’s esophagus, and glaucoma. His medications included hydrochlorothiazide 25 mg daily, bisoprolol 10 mg daily, lisinopril 20 mg daily, simvastatin 20 mg daily, pantoprazole 20 mg daily, and timolol ophthalmic 0.5% one drop in each eye twice daily. The review of systems was otherwise negative. On examination, he was hemodynamically stable and in no distress with blood pressure (BP) of 135/60 mmHg and a heart rate (HR) of 61 beats per minute. The remainder of his examination was unremarkable. After reviewing each of his medications, we discovered that his ophthalmologist had added timolol ophthalmic drops several weeks prior. He then realized that the episodes had begun occurring after he had started using these eye drops. We recommended that he hold the use of the eye drops for three days and evaluate for the association of symptoms. The patient reported back that the episodes stopped upon temporary cessation of the eye drops.

## Discussion

Ophthalmic topical medications are highly concentrated and are often inadvertently absorbed systemically due to the passage of medication through the nasolacrimal duct into the highly vascular nasal mucosa [6]. Ophthalmologic drops are often neglected when obtaining medication reconciliation and considering adverse drug reactions. Due to the high incidence of glaucoma and the frequent use of eye drops as first-line therapy, it is important for providers to be familiar with the indication, the active ingredients, and the potential systemic side effects of these medications. There are five medication classifications of topical eye drops used to treat glaucoma: beta-blockers, prostaglandin analogs, alpha-adrenergic agonists, carbonic anhydrase inhibitors, and cholinergic agents (Table 1). As with the treatment for systemic hypertension, a combination of the medication classes is frequently required [4].

Beta-blocker eye drops, such as timolol, were developed in the late 1970s after a cardiologist, William Frishman, MD, observed lower intraocular pressures in patients on oral beta-blockers. It was discovered that this medication reduced the production of aqueous fluid in the eye, thereby lowering intraocular pressure. For years, beta-blockers were considered first-line therapy. They are long-acting and have a low incidence of ocular side effects; however, they have been noted to have similar systemic adverse effects as oral therapies, including drowsiness, hypotension, bradycardia, syncope, shortness of breath, bronchospasm and status asthmaticus, fatigue, chest pain, and impotence. This class of oral medications should be used cautiously in patients with a history of cardiac disease and is relatively contraindicated in patients with asthma and chronic obstructive pulmonary disease [3].

Prostaglandin analogs, such as latanoprost or travoprost, are often the most effective topical agent at reducing intraocular pressure and are now frequently used as first-line therapy [3,7]. Prostaglandins improve aqueous outflow, thereby reducing intraocular pressure. They have demonstrated better medication tolerance. Rare systemic adverse effects have been reported, including angina, upper respiratory infection, and muscle or joint pain. Few local side effects have been reported as well, including mild conjunctival injection and burning of the eyes, blurry vision, increased pigmentation of the iris, and increased growth of the eyelashes.

Although used less commonly for the treatment of glaucoma, there are several other ophthalmic glaucoma medications that have important potential adverse effects. Alpha-adrenergic agonists, such as brimonidine, work by decreasing aqueous humor production; however, they may cause dry mouth, headache, fatigue, and, less frequently, anxiety, dizziness, arrhythmia, and abnormal taste.

Another line of medications includes carbonic anhydrase inhibitors, such as brinzolamide, which decrease aqueous humor production. These are structurally related to sulfonamides and should be avoided in patients who have sulfa allergies as they can pose severe systemic adverse effects including Stevens-Johnson syndrome, malaise, and renal calculi [3,6]. These frequently lead to bitter or metallic taste and, in rare cases, punctate keratitis.

Lastly, there is a class of medications called cholinergic agents, such as pilocarpine, which increase outflow through the trabecular meshwork. The most frequently reported side effects include headache, urinary retention, small pupils, and blurred vision.

Perhaps, contrary to popular belief, ophthalmologic medications have the potential to lead to significant systemic adverse effects due to the highly concentrated doses and direct absorption via the highly vascular nasal mucosa after passage through the lacrimal duct. Patients on multiple medications are more likely to experience adverse effects [8]. This route of absorption bypasses the liver. There are two proposed techniques that may reduce systemic absorption. The first technique simply involves closing the eye following the administration of the eye drops for two minutes. The second technique, known as punctal occlusion, requires patients to use either a silicon lacrimal plug or place a finger in the corner of the eye to gently occlude the lacrimal duct for three minutes. Multiple studies have shown that this latter method improves the therapeutic index of various glaucoma medications [9,10,11]. Both methods reduce but do not eliminate dose-related effects from systemic absorption [12].

**Table 1:** Topical eye drops for treating glaucoma

Class	Medications	Mechanism	Common side effects
Beta-blockers	Timolol, levobunolol HCI, metipranolol, betaxolol HCI	Reduce the production of aqueous fluid in the eye	Drowsiness, hypotension, bradycardia, syncope, shortness of breath, bronchospasm and status asthmaticus, fatigue, chest pain, impotence
Prostaglandin analogs	Latanoprost, travoprost, tafluprost, bimatoprost	Improve aqueous outflow from the eye	Angina, upper respiratory infection, muscle or joint pain, conjunctival injection, burning of the eyes, blurry vision, increased pigmentation of the iris, increased growth of the eyelashes
Alpha-adrenergic agonists	Brimonidine, apraclonidine HCI	Decrease aqueous humor production	Dry mouth, headache, fatigue, and, less frequently, anxiety, dizziness, arrhythmia, abnormal taste
Carbonic anhydrase inhibitors	Brinzolamide, methazolamide, dorzolamide HCI, acetazolamide	Decrease aqueous humor production	Stevens-Johnson syndrome, blurred vision, eye irritation, eye pain, eye discharge, itchy eye, dry eye, abnormal eye sensation, redness of the eye, malaise, renal calculi, bitter or metallic taste, punctate keratitis
Cholinergic agents	Pilocarpine HCl, carbachol	Increase outflow through the trabecular meshwork	Headache, urinary retention, small pupils, blurred vision

## Conclusions

As glaucoma is a leading cause of vision loss in the US and eye drops are most commonly prescribed as the mainstay of treatment, there is an area of opportunity for providers to prevent potentially devastating, at the least, very unnerving, side effects. It is recommended that providers encourage patients to perform the punctal occlusion or eye closure technique when administering the drops, particularly those who have already experienced side effects. Due to the potential for systemic absorption through the nasopharyngeal mucosa, drug-drug interactions must always be considered when prescribing a new oral medication to a patient who is also using topical ophthalmologic drops and vice versa. If a patient cannot tolerate treatment due to adverse reactions or poor compliance or develops progressive vision loss refractory to treatment, then other medications or types of therapy (e.g., laser procedure, surgery) should be considered. It is imperative that patients be educated on common systemic side effects of such drugs when they are prescribed.
